# Periostin drives extracellular matrix degradation, stemness, and chemoresistance by activating the MAPK/ERK signaling pathway in triple–negative breast cancer cells

**DOI:** 10.1186/s12944-023-01912-1

**Published:** 2023-09-16

**Authors:** Jinna Wu, Jia Li, Huiya Xu, Ni Qiu, Xiaojia Huang, Hongsheng Li

**Affiliations:** 1 Department of Breast Oncology Surgery, Affiliated Cancer Hospital & Institute of Guangzhou Medical University, Guangzhou, 510095 China; 2 Department of Pathology, Sun Yat-sen Memorial Hospital, Sun Yat-sen University, Guangzhou, 510120 China

**Keywords:** Triple**–**negative breast cancer, Adipocytes, Palmitic acid, Periostin, MAPK/ERK signaling pathway

## Abstract

**Background:**

Adipose tissue, which is mainly composed of adipocytes, is a crucial component of the tumor microenvironment, particularly in breast cancer. Adipocytes surround breast cancer cells and may participate in cell‒cell interactions in the breast microenvironment. However, little is currently known about how adipocytes influence the biological behavior of the surrounding breast cancer cells. Hence, this study sought to investigate the role and underlying mechanisms of periostin in triple–negative breast cancer (TNBC) cells cocultured with adipogenic conditioned medium (ACM) and palmitic acid (PA).

**Methods:**

Human TNBC cell lines (MDA‒MB‒231 and SUM159PT) were treated with ACM and PA, then the expression of periostin, matrix metalloproteinases (MMPs) and stemness**–**related molecules were assessed by Western blotting and RT‒qPCR. The cellular viability was assessed using CCK‒8 assay. Plasmid transfection, RNA sequencing, and pathway inhibitor were used to explore the specific mechanisms of periostin.

**Results:**

ACM and PA elevated the expression of both MMPs and stemness**–**related molecules in TNBCs. MMPs can promote tumor cell infiltration and migration by degrading the extracellular matrix, and stemness expression increases the development of tumor chemoresistance. Additionally, ACM and PA increased periostin expression, while inhibiting periostin disrupted the involvement of ACM and PA in promoting extracellular matrix degradation, stemness, and chemoresistance in TNBCs. Furthermore, this study found that periostin promoted TNBC progression by activating the MAPK/ERK signaling pathway and that inhibition of MAPK/ERK signaling reduced the phenotype caused by periostin upregulation in TNBCs treated with ACM or PA. Finally, the present results showed that the high expression of POSTN, which encodes periostin, was substantially related to worse survival in TNBC patients.

**Conclusions:**

The results of the study elucidated for the first time how periostin is the key protein secreted in TNBCs in response to the adipocyte**–**regulated tumor microenvironment, while periostin**–**neutralizing antibodies may serve as potential therapeutic agents in relation to TNBC progression.

**Supplementary Information:**

The online version contains supplementary material available at 10.1186/s12944-023-01912-1.

## Introduction

Breast cancer has become the most common cancer in women and the main cause of death from cancer worldwide [1]. Triple**–**negative breast cancer (TNBC) accounts for approximately 10–24% of all breast cancer cases, and it is distinguished by a lack of estrogen receptor (ER), progesterone receptor (PR), and human epidermal growth factor receptor 2 (HER2) expression [2]. Due to the lack of response to endocrine therapy or HER2-targeted treatment, systemic chemotherapy is the standard drug treatment for TNBC patients, who frequently face the issues of chemoresistance and metastatic recurrence [3]. Thus, there is an urgent need to investigate the molecular mechanism associated with TNBC to improve current concepts of disease prevention and therapy.

The tumor microenvironment potentially plays a regulatory role in the development, progression, and metastasis of many malignancies. More specifically, different types of cells, including adipocytes, endothelial cells, and immune cells, within the tumor microenvironment continuously interact with cancer cells, thereby playing a significant role in tumor growth and progression [4, 5]. Adipocytes comprise approximately 90% of breast tissue. Moreover, crosstalk between adipocytes and cancer cells leads to the involvement of adipocytes in breast cancer development, metastasis, and treatment resistance [6]. However, both the interactions between adipocytes and breast cancer cells within the microenvironment and the underlying mechanisms are complicated and remain unclear [7].

Periostin, which is encoded by the POSTN gene, is both an extracellular matrix protein and a secreted protein [8]. It has been linked to various types of cancers, most notably breast cancer, and has been found to be functionally involved in multiple steps of malignant development, including epithelial**–**mesenchymal transition, angiogenesis, and metastasis [9, 10]. Clinical studies have demonstrated that periostin upregulation (or elevated circulating levels of periostin) is associated with an increased metastatic tumor burden and a poor patient prognosis [11, 12].

However, there have been no reports concerning the role and underlying molecular mechanisms of periostin in relation to communication between adipocytes and TNBC cells (TNBCs). Hence, in this study, we sought to examine the expression, role, and underlying mechanisms of periostin in TNBCs cocultured with adipogenic conditioned medium (ACM) and palmitic acid (PA) to gain insight into the mechanisms behind crosstalk between adipocytes and breast cancer cells as well as to identify effective targets for TNBC treatment.

## Materials and methods

### Cell culture

Human TNBC cell lines (MDA**–**MB**–**231 and SUM159PT) were purchased from American Type Culture Collection (ATCC, Manassas, USA). Cells were cultured in RPMI 1640 medium (Gibco, Life Technologies, New York, USA) containing 10% fetal bovine serum (Gibco, Life Technologies, New York, USA) in a humidified incubator with 5% carbon dioxide and 37 °C.

For the in vitro experiments, PA (GLPBIO, Montclair, USA, 300 µM), recombinant human periostin (novoprotein, Shanghai, China, 1 µg/ml), periostin neutralizing antibodies (R&D, Minneapolis, USA, 1:1000), and U0126**–**EtOH (GLPBIO, Montclair, USA, 10 µM) were purchased from the indicated companies and administered at the indicated concentrations.

### Differentiation and collection of ACM

This study was approved by the Medical Ethics Committee of Affiliated Cancer Hospital & Institute of Guangzhou Medical University. Moreover, signed informed consent forms were obtained from each subject. We collected adipose tissue from the breast cancer mastectomy specimens of five TNBC patients. The participating patients’ clinical and pathological information is listed in Supplementary Table S1.

Mature human adipocytes were obtained from human adipose tissue within the breast cancer mastectomy specimens. Adipose tissue**–**derived adipocytes were prepared as previously described [13]. Briefly, fresh adipose tissue was dissected and minced. The adipose tissue pieces were digested with 1 mg/ml collagenase II in a shaking incubator at 37 °C for 1 h. Subsequently, the digestion solution was filtered through a 100 μm cell sieve. The primary preadipocytes were then maintained in DMEM/F12 (Gibco, Life Technologies, New York, USA) containing 10% fetal bovine serum (Gibco, Life Technologies, New York, USA) and 1% penicillin**–**streptomycin (Gibco, Life Technologies, New York, USA) before being cultured in differentiation medium to obtain mature adipocytes, which were then cultured in maintenance medium for 24 h to collect the adipogenic conditioned medium. The differentiation medium contains the following ingredients: serum**–**free DMEM/F12, 2.5 mM glutamine, 15 mM HEPES, 1% antibiotic**–**antimycotic, 10 mg/ml transferrin, 33 µM biotin, 0.5 µM human insulin, 17 µM pantothenate, 0.1 µM dexamethasone, 2 nM T3, 540 µM IBMX, and 1 µM ciglitazone. The maintenance medium contained serum**–**free DMEM/F12, 2.5 mM glutamine, 15 mM HEPES, 1% antibiotic**–**antimycotic, 10 mg/ml transferrin, 33 µM biotin, and 0.5 µM human insulin. For the in vitro experiments, the TNBCs were treated with 100% ACM for 24 h, and the experiments were performed using at least three biological replicates.

### Real‒time quantitative polymerase chain reaction (RT‒qPCR)

Total RNA was extracted using TRIzol reagent (Invitrogen, Carlsbad, USA) and reverse transcribed into cDNA with a PrimeScript kit (Thermo Scientific, Waltham, USA) according to the manufacturer’s protocol. RT‒qPCR was performed with the SYBR^®^ Green Kit (Thermo Scientific, Waltham, USA) in an ABI 7500 Real**–**Time PCR System (Applied Biosystems, USA). β**–**actin was used as an internal control. Relative mRNA expression was calculated using the 2^−ΔΔCT^ method. The primer sequences are listed in Table 1.

**Table 1:** Primers for qPCR

Gene	Primer sequence (5’- 3’)
β**–**actin	Forward: CATGTACGTTGCTATCCAGGC Reverse: CTCCTTAATGTCACGCACGAT
*POSTN*	Forward: GCTATTCTGACGCCTCAAAACT Reverse: AGCCTCATTACTCGGTGCAAA
*MMP2*	Forward: TACAGGATCATTGGCTACACACC Reverse: GGTCACATCGCTCCAGACT
*MMP9*	Forward: AGACCTGGGCAGATTCCAAAC Reverse: CGGCAAGTCTTCCGAGTAGT
*MMP13*	Forward:TCCTGATGTGGGTGAATACAATG Reverse: GCCATCGTGAAGTCTGGTAAAAT
*MYC*	Forward: GGCTCCTGGCAAAAGGTCA Reverse: CTGCGTAGTTGTGCTGATGT
*SOX2*	Forward: TGGACAGTTACGCGCACAT Reverse: CGAGTAGGACATGCTGTAGGT
*NANOG*	Forward: CCCCAGCCTTTACTCTTCCTA Reverse: CCAGGTTGAATTGTTCCAGGTC

### Immunoblotting

Total protein was extracted from cells using RIPA buffer (Beyotime, Shanghai, China) in the presence of a protease inhibitor mixture (Pierce Chemical, Dallas, Texas, USA) and quantified using a BCA protein assay kit (Thermo Scientific, Waltham, USA). Western blotting was performed according to standard procedures. The following primary antibodies were used: anti**–**periostin (Proteintech, #66,491**–**1**–**lg, 1:3000), anti**–**ERK1/2 (CST, #9102, 1:1000), anti**–**phospho ERK1/2 (CST, #4370, 1:1000), anti**–**P38 (CST, #9212, 1:1000), anti**–**phospho P38 (CST, #9215, 1:1000), and anti**–**β**–**actin (Affinity Biosciences, #AF7018, 1:1000).

### Enzyme–linked immunosorbent assay (ELISA)

The conditioned medium (CM) of TNBCs cultured under different conditions was collected, and the concentration of periostin was determined with a human ELISA kit (Boster, California, USA) according to the manufacturer’s protocols.

### Cell proliferation assay

To determine the proliferation of cells under different culture conditions, cell proliferation assays were performed using a CCK**–**8 kit (Beyotime, Shanghai, China) according to the manufacturer’s instructions. In brief, the cells were seeded at a density of either 1 × 10^3^ or 1 × 10^4^ cells/well in a 96**–**well plate according to the specific experimental situation. The absorbance at 450 nm was determined using a BioTek Synergy 2 system, and the relative cell proliferation was calculated according to the formula included in the kit.

### Cell transfection

To establish cells transfected with POSTN knockdown, Lipofectamine 3000 (Invitrogen, Carlsbad, USA) was used to transfect cells with psi**–**LVRU6GP vectors containing specific shRNAs (GeneChem, Shanghai, China). The shRNA**–**POSTN sequence (F: CGGTGACAGTATAACAGTAAA, R: CGGTGACAGTATAACAGTA AA) was used for POSTN downregulation.

### RNA sequencing and analysis

Total RNA was extracted from MDA**–**MB**–**231 cells treated with IgG antibody or rhPeriostin, and libraries were constructed using the VAHTS Stranded mRNA**–**seq Library Prep Kit for Illumina v2. The quality of the library was checked using Qubit 2100, and the concentrations were determined according to the analysis profiles. Genes expressed at a log_2_ (fold change) > 1 or < **− 1** and a *P* < 0.05 were identified as differentially expressed genes and then subjected to Gene Ontology (GO) enrichment analysis and Kyoto Encyclopedia of Genes and Genomes (KEGG) pathway analyses using DAVID Bioinformatic Resources 6.8.

### UALCAN and Kaplan‒Meier plotter

UALCAN (http://ualcan.path.uab.edu/) is a comprehensive, interactive web resource for analyzing cancer omics data [14]. In this study, the expression of POSTN in breast cancer was analyzed using UALCAN based on the major subclasses. The expression level of POSTN was normalized as the transcripts per million reads. The Kaplan‒Meier plotter online database (http://kmplot.com) is capable of assessing the association between genes and survival in many types of cancer samples, including breast cancer, ovarian cancer, lung cancer, and gastric cancer samples. Kaplan‒Meier plotter was applied to illustrate the prognostic value of POSTN at the mRNA level based on cases enrolled from The Cancer Genome Atlas (TCGA). In terms of generating a Kaplan‒Meier curve, a convenient and widespread option for determining the cutoff is the median expression value [15, 16]. In this study, the median expression value was used to separate the POSTN**–**low and POSTN**–**high groups based on the Kaplan‒Meier curve. Moreover, various prognostic values, including relapse**–**free survival (RFS), overall survival (OS), and distant metastasis**–**free survival (DMFS) of POSTN, were evaluated in TNBC.

### Statistical analyses

All data were analyzed by SPSS v13.0 software and presented as the mean ± standard deviation (SD). Statistical significance was considered by the two**–**tailed independent Student’s *t* test for comparing two independent groups and by one**–**way analysis of variance (ANOVA) for multiple comparisons. In this study, the experiments were performed with at least three biological replicates, and a *P* value < 0.05 was considered statistically significant.

## Results

### ACM and PA increase the expression of matrix metalloproteinases and stemness–related molecules in TNBC cells

**Fig. 1:**
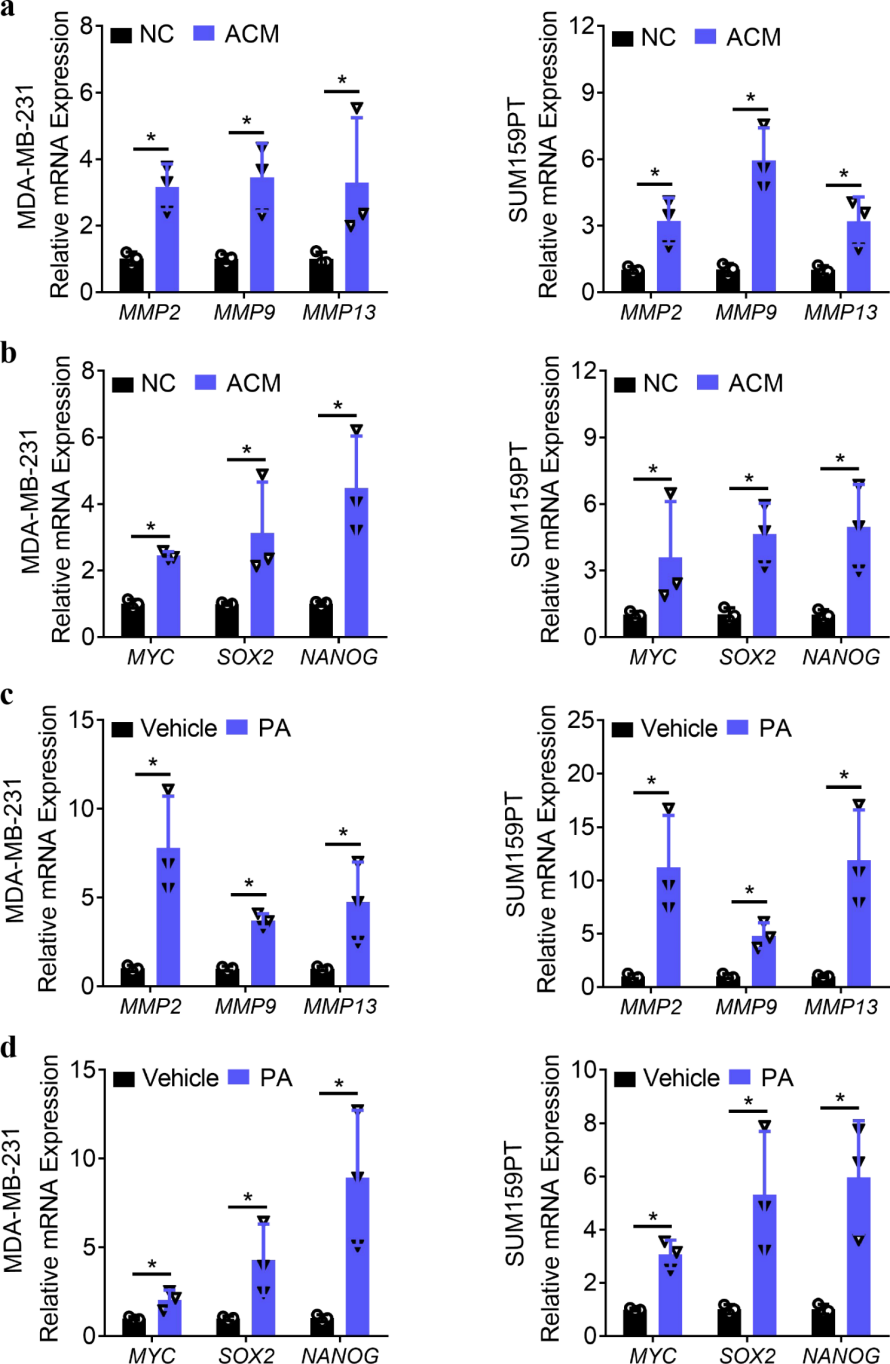
ACM and PA increase the expression of MMPs and stemness–related molecules in TNBCs. **a–b.** qPCR analysis of the expression of MMPs (*MMP2, MMP9*, and *MMP13*) (a) and stemness**–**related molecules (*MYC, SOX2*, and *NANOG*) (b) in TNBCs (MDA**–**MB**–**231 and SUM159PT cell lines) treated with NC or ACM for 24 h. **c–d.** qPCR analysis of the expression of MMPs (c) and stemness**–**related molecules (d) in MDA**–**MB**–**231 and SUM159PT cells treated with vehicle or PA for 24 h. NC: negative control, ACM: adipogenic conditioned medium. *P < 0.05

To determine the effect of human primary adipocytes in TNBC, the TNBC cell lines MDA**–**MB**–**231 and SUM159PT were cocultured with ACM for 24 h. Subsequently, the expression of matrix metalloproteinases (MMPs) (*MMP2*, *MMP9*, and *MMP13*) and stemness**–**related molecules (*MYC*, *SOX2*, and *NANOG*) [17–20] was determined to be significantly upregulated in the TNBCs (Fig. 1a–b), which demonstrated that ACM promoted the degradation of the extracellular matrix and stemness of the TNBCs. High**–**fat diets are rich in saturated fatty acids, including palmitic acid (PA), a 16**–**carbon long**–**chain saturated fatty acid commonly found in dietary fats. Therefore, we also sought to explore the role of PA in TNBCs. To accomplish this, the TNBCs were treated with 300 µM/mL PA for 24 h, and PA also significantly increased the expression of MMPs as well as stemness**–**related molecules in the TNBCs (Fig. 1c–d). These results suggest that ACM and PA have important effects on the biological functions of TNBCs.

### ACM and PA induce the expression and secretion of periostin in TNBCs

**Fig. 2:**
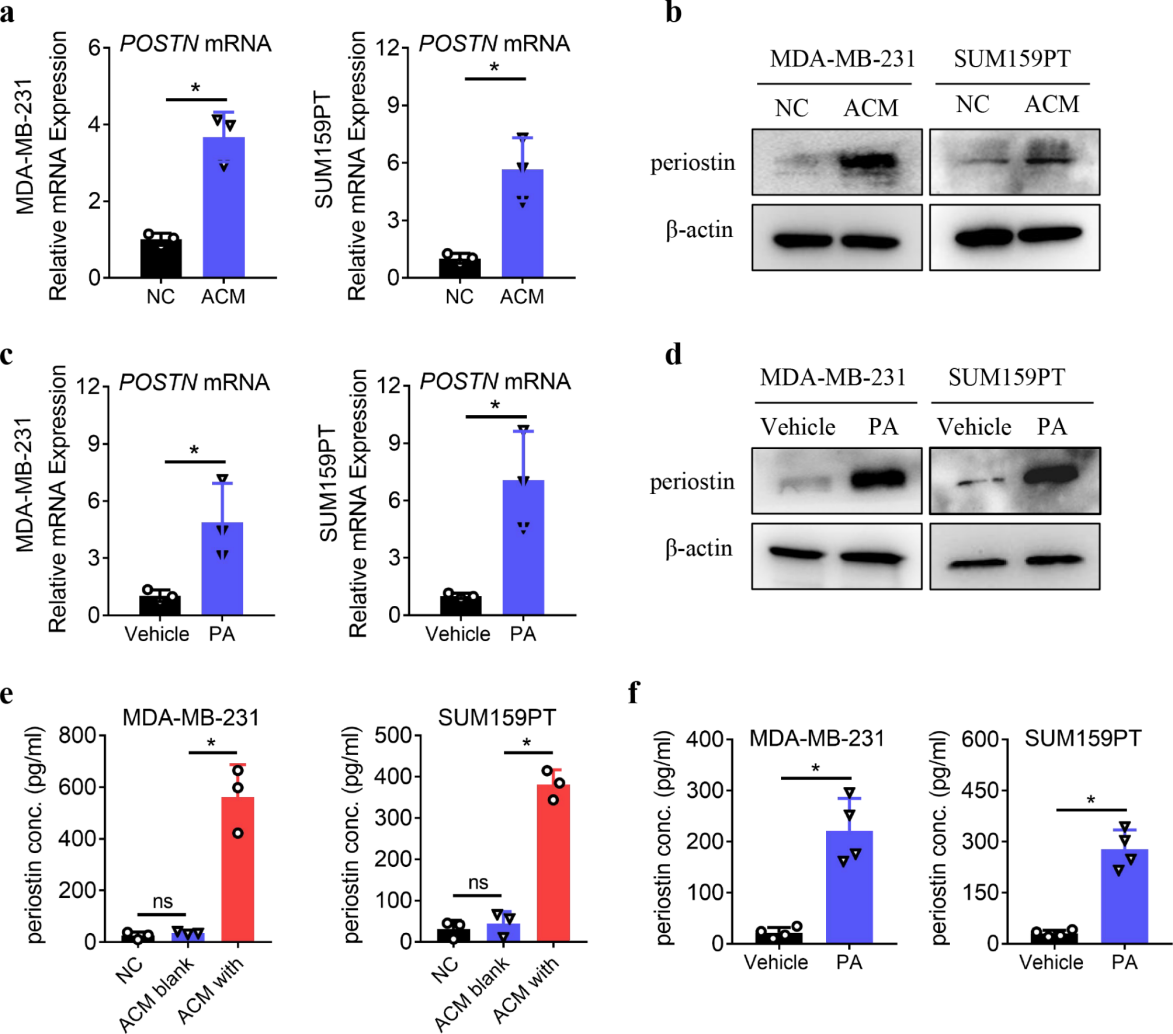
ACM and PA induce the expression and secretion of periostin in TNBCs. **a–b.** MDA**–**MB**–**231 and SUM159PT cells were treated with NC or ACM for 24 h, and then the *POSTN* mRNA and protein levels were determined by qPCR (a) and Western blotting (b) analysis. **c–d.** MDA**–**MB**–**231 and SUM159PT cells were treated with vehicle or PA for 24 h, and then the *POSTN* mRNA and protein levels were determined by qPCR (c) and Western blotting (d) analysis. **e–f.** The concentration of periostin in the cell culture medium was determined by ELISA. NC: negative control; ACM blank: ACM alone; ACM with: ACM cocultured with TNBCs. ns: no significance; *P < 0.05

In further experiments, we also found that the expression of *POSTN* mRNA and periostin protein was upregulated in TNBCs treated with ACM and PA (Fig. 2a–d). Additionally, ELISA was performed to determine the secretory levels of periostin, a secretory protein encoded by the POSTN gene. The ELISA results indicated that the levels of periostin in the supernatant of the negative control and ACM alone samples were very low; however, when ACM was cocultured with TNBCs, the secretion of periostin in the supernatant was dramatically higher (Fig. 2e), suggesting that the interaction between the ACM and TNBCs stimulated periostin secretion from the TNBCs. In addition, periostin secretion was also significantly elevated when TNBCs were treated with PA (Fig. 2f).

### Inhibition of periostin restores the cellular processes in TNBCs

**Fig. 3:**
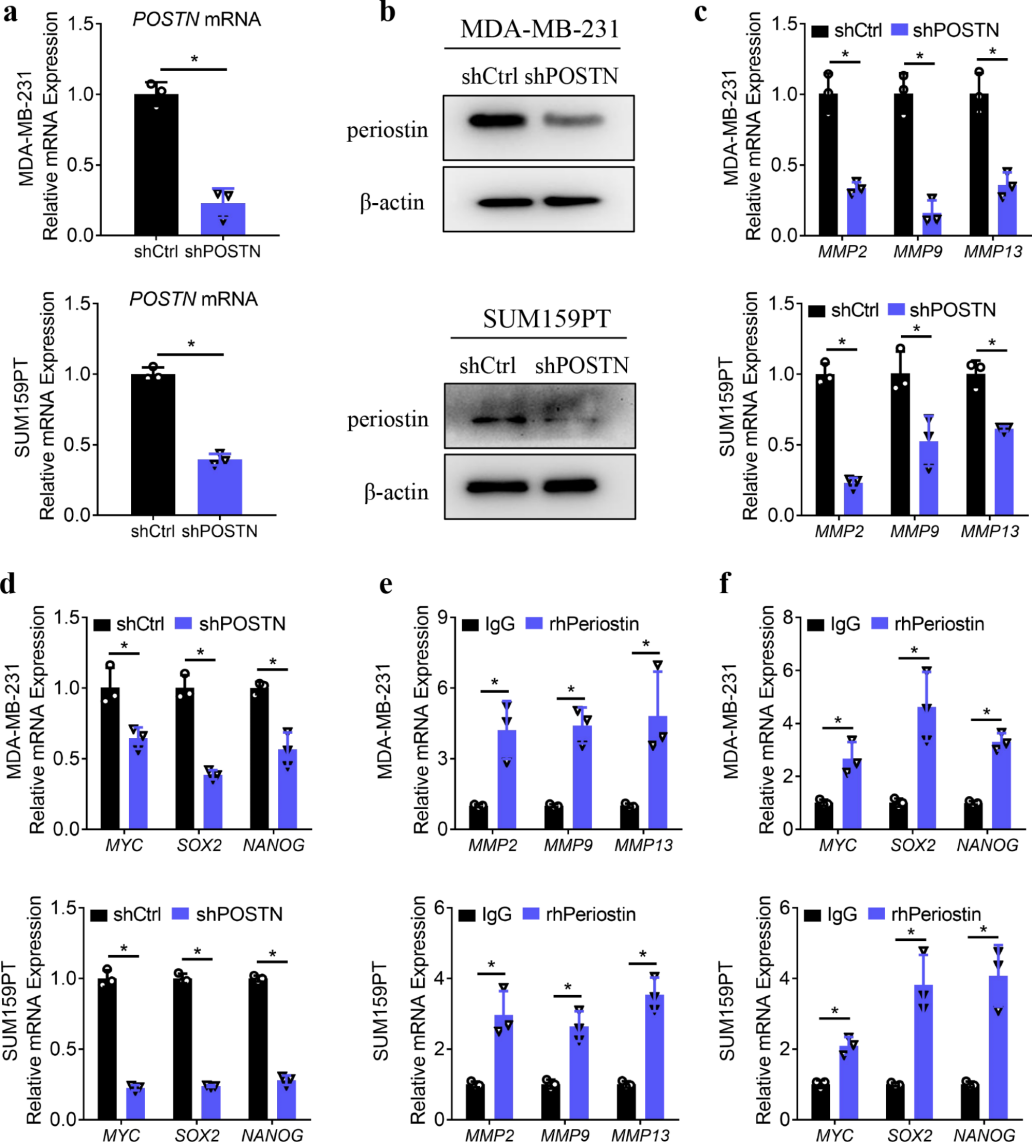
Inhibition of periostin restores the cellular processes in TNBCs. **a–b.** The results of the qPCR (a) and Western blotting (b) analysis of the specificity of shRNA against POSTN in MDA**–**MB**–**231 and SUM159PT cells, where β**–**actin was used as the control. **c–d.** The results of the qPCR analysis of the MMPs (*MMP2, MMP9*, and *MMP13*) (c) and stemness–related molecules (*MYC, SOX2*, and *NANOG*) (d) in MDA**–**MB**–**231 and SUM159PT cells transfected with shPOSTN or shCtrl for 48 h. **e–f.** The results of the qPCR analysis of the MMPs (e) and stemness**–**related molecules (f) in MDA**–**MB**–**231 and SUM159PT cells treated with rhPeriostin or IgG for 24 h. *P < 0.05

Given that ACM and PA could regulate the expression of periostin, we wondered whether the regulation of periostin would affect the role of ACM and PA in TNBCs. Thus, we transfected POSTN**–**specific shRNA (shPOSTN) into MDA**–**MB**–**231 and SUM159PT cells. The results of the qPCR and Western blotting analyses showed that shRNA effectively inhibited periostin expression in both cell lines at the RNA and protein levels (Fig. 3a–b). We then examined whether the inhibition of periostin affects the cellular processes in TNBCs. qPCR analysis revealed decreased expression of MMPs and stemness**–**related molecules in TNBCs transfected with shPOSTN compared with the cells transfected with the control shCtrl (Fig. 3c–d). In addition, when we treated TNBCs with recombinant human periostin protein (rhPeriostin) for 24 h, the expression of MMPs and stemness**–**related molecules increased significantly (Fig. 3e–f). These results suggest that periostin is regulated by ACM and PA and that the inhibition of periostin affects the cellular processes of extracellular matrix degradation and stemness formation in TNBCs.

**Fig. 4:**
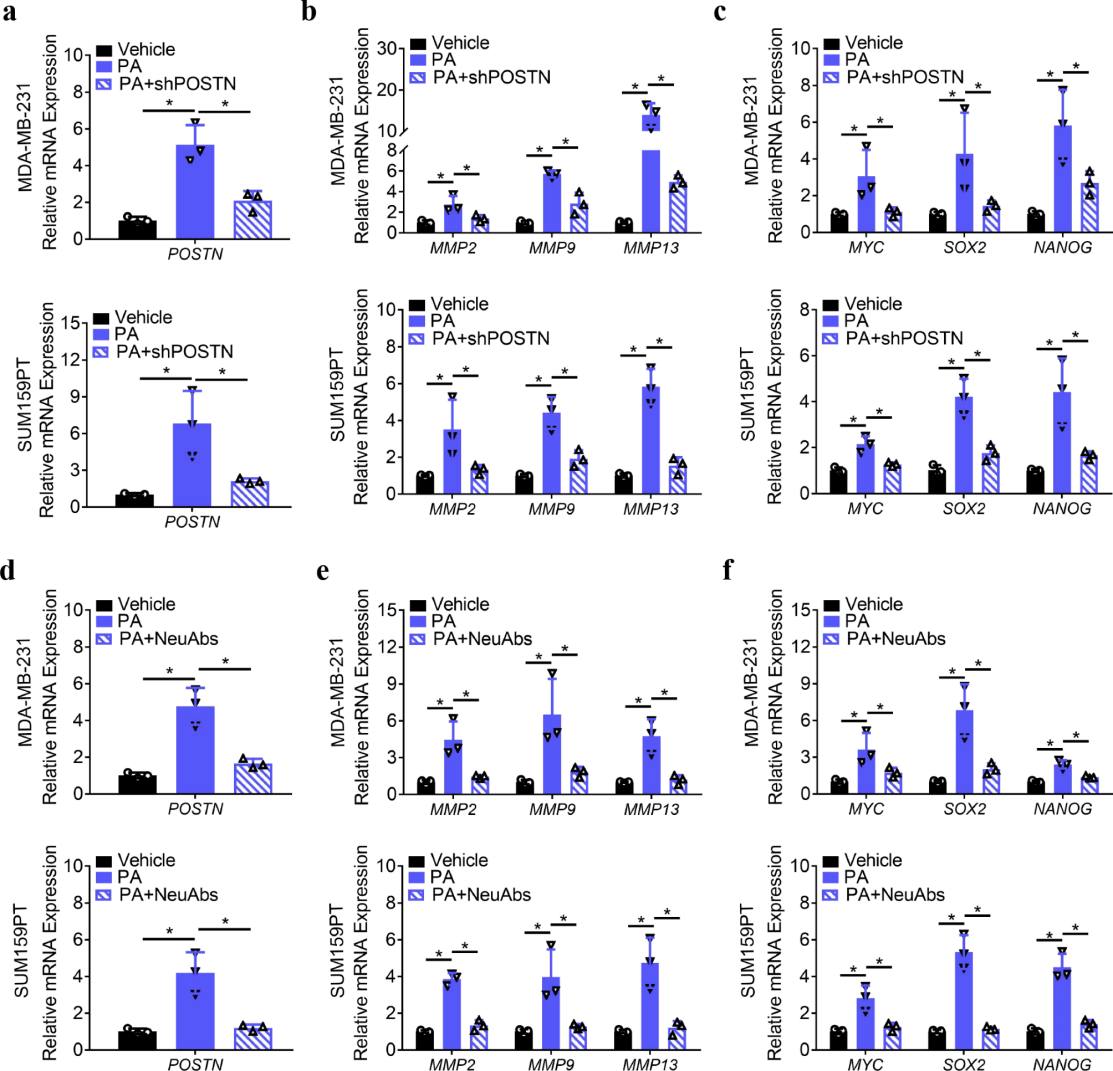
ACM and PA function in TNBCs by regulating the expression and secretion of periostin. **a–c.** MDA**–**MB**–**231 and SUM159PT cells were treated with vehicle or PA for 24 h after transfection with shPOSTN, and then the related gene expression was detected by qPCR. **d–f.** MDA**–**MB**–**231 and SUM159PT cells were treated with vehicle or PA and/or periostin NeuAbs for 24 h, and then the related gene expression was detected by qPCR. *P < 0.05

Moreover, we sought to reveal whether the role of ACM and PA in TNBCs was mediated by periostin. Hence, MDA**–**MB**–**231 and SUM159PT cells were treated with ACM or PA following POSTN knockdown via shPOSTN and periostin inactivation via periostin**–**neutralizing antibodies (NeuAbs) [21, 22]. Subsequently, the changes in the expression of MMPs and stemness**–**related molecules were identified by qPCR analysis. Our experimental results demonstrated that periostin knockdown significantly reversed the PA**–**induced facilitation of MMPs and stemness**–**related molecule expression in TNBCs (Fig. 4a–c). In addition, when PA acted on TNBCs while periostin was neutralized using NeuAbs for 24 h, the PA**–**induced increase in the expression of MMPs and stemness**–**related molecules in the TNBCs was strongly inhibited (Fig. 4d–f). Collectively, these results suggest that periostin is involved in changes in the cellular processes of ACM**–** and PA**–**induced extracellular matrix degradation and stemness formation in TNBCs.

### Periostin promotes the tolerance of TNBCs toward paclitaxel chemotherapy

**Fig. 5:**
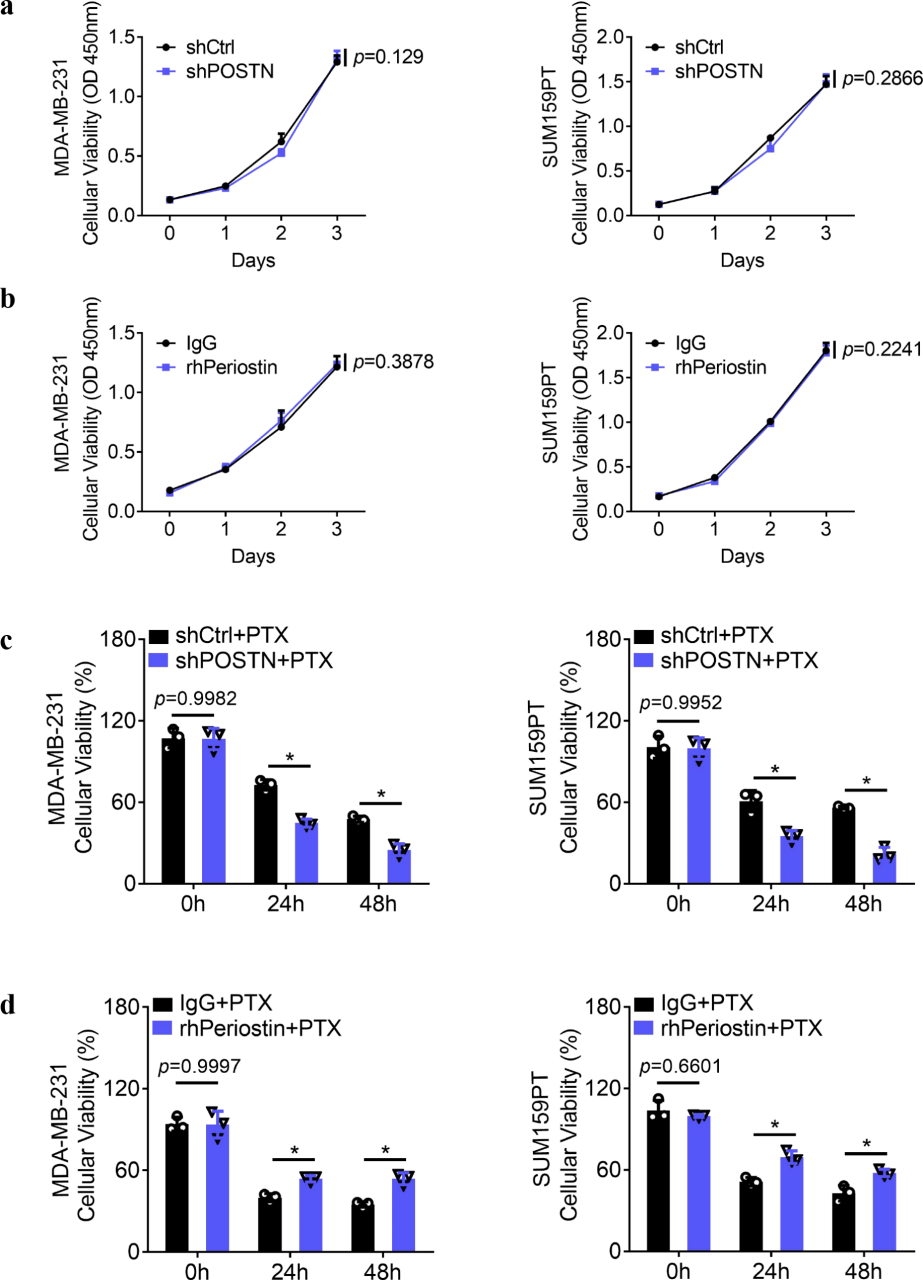
Periostin promotes the tolerance of TNBCs toward paclitaxel chemotherapy. **a–b.** A CCK**–**8 assay was used to determine cell proliferation in MDA**–**MB**–**231 and SUM159PT cells under the indicated conditions. **c–d.** A CCK**–**8 assay was used to determine the cellular viability when MDA**–**MB**–**231 and SUM159PT cells were treated with 3 µg/ml PTX under the indicated conditions. *P < 0.05

Furthermore, we wondered whether periostin plays a role in the chemoresistance of TNBCs. To address this issue, we performed a CCK**–**8 assay to test the tolerance of TNBCs to the first-line chemotherapeutic agent paclitaxel (PTX) in MDA**–**MB**–**231 and SUM159PT cells transfected with shPOSTN or treated with rhPeriostin. The CCK**–**8 results showed that changes in periostin expression had no significant effect on the proliferation of TNBCs (Fig. 5a–b); however, inhibition of periostin expression enhanced the sensitivity of TNBCs to PTX treatment and significantly reduced tumor cell viability, whereas the killing effect of PTX on TNBCs was greatly diminished when rhPeriostin was administered (Fig. 5c–d). These results suggest that periostin does not affect cell viability in TNBCs, but it significantly affects the tolerance of TNBCs toward chemotherapeutic agents.

### Periostin activates the MAPK signaling pathway to promote extracellular matrix degradation, stemness, and chemoresistance in TNBCs

**Fig. 6:**
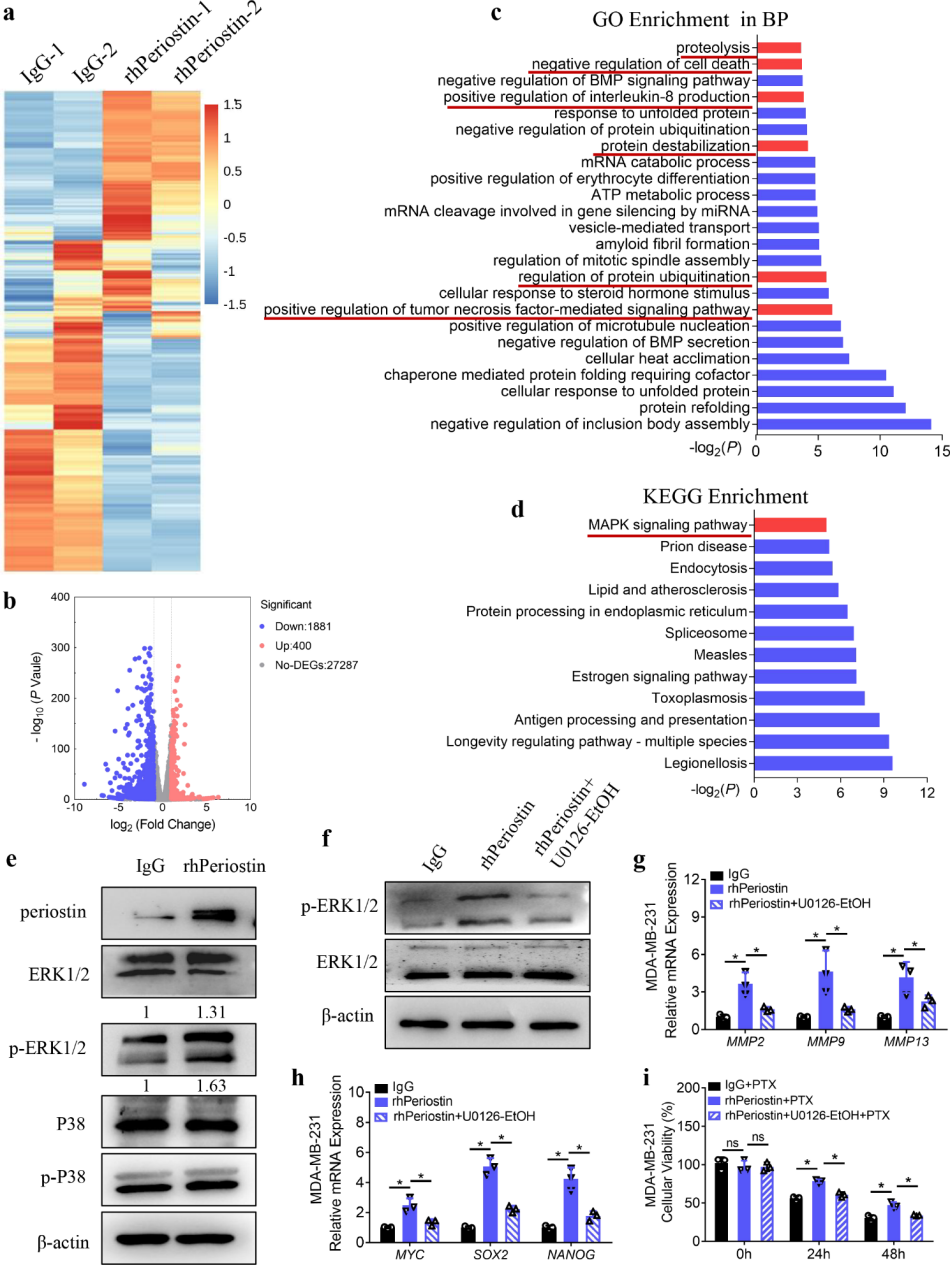
Periostin activates the MAPK signaling pathway to promote extracellular matrix degradation, stemness, and chemoresistance in TNBCs. **(a)** Four samples representative of the transcriptome heatmap. **(b)** Volcano plot of the RNA**–**seq results. **c–d.** GO term enrichment analysis in the BP category (c) and KEGG pathway enrichment analysis (d) of the upregulated genes in the rhPeriostin group compared with the IgG group. The bars marked in red were the ones we focused on. **e–f.** Western blotting analysis of the indicated protein levels in MDA**–**MB**–**231 cells treated with IgG or rhPeriostin and/or U0126**–**EtOH for 24 h. The densitometry results of p**–**ERK1/2 (upper and bottom strips) were expressed as fold change in the protein levels when compared with IgG**–**treated MDA**–**MB**–**231 cells after being normalized to β**–**actin (e). **g–h.** qPCR analysis of the indicated gene expression in MDA**–**MB**–**231 cells treated with IgG or rhperiostin and/or U0126**–**EtOH for 24 h. **i.** CCK**–**8 analysis of the cellular viability when MDA**–**MB**–**231 cells were treated with 3 µg/ml PTX in the indicated conditions. *P < 0.05

We further explored the underlying molecular mechanisms whereby periostin enhances extracellular matrix degradation, stemness, and chemoresistance in TNBCs. First, RNA from MDA**–**MB**–**231 cells treated with rhPeriostin for 24 h was collected for the purpose of RNA sequencing (RNA**–**seq). The RNA**–**seq results showed that 400 genes were significantly upregulated and 1881 genes were significantly downregulated in the rhPeriostin group compared with the control IgG group (Fig. 6a–b). Next, the differentially expressed genes with a fold change (FC) > 2 and a *P* < 0.05 were analyzed by GO term enrichment analysis in the biological processes (BP) category and KEGG pathway enrichment analysis. The GO term mapping of the upregulated genes in the rhPeriostin group showed that proteolysis, the negative regulation of cell death, the regulation of protein ubiquitination, and the positive regulation of the tumor necrosis factor**–**mediated signaling pathway were upregulated (Fig. 6c), which further validated our abovementioned finding that periostin upregulates the expression of MMP**–**related molecules and chemoresistance. A growing body of evidence suggests that MMPs, the major family of proteases associated with tumorigenesis, could alter proteolysis, protein ubiquitination and protein degradation to facilitate tumor growth, tissue invasion and metastasis [23, 24]. The negative regulation of cell death meant that the cells were more viable when subjected to changes in the external environment, which was consistent with our finding that TNBCs are tolerant of PTX treatment when rhPeriostin is included in the culture medium. Meanwhile, the results of the KEGG pathway enrichment analysis indicated that the MAPK signaling pathway was highly activated (Fig. 6d).

To confirm the increased activity of the MAPK signaling pathway in the rhPeriostin group, Western blotting analysis was performed on MAPK signaling pathway**–**related molecules. The results revealed no significant difference in the level of phosphorylation of p38 (p**–**P38) but a significant increase in the level of phosphorylation of ERK1/2 (p**–**ERK1/2) in rhPeriostin**–**treated MDA**–**MB**–**231 cells compared with the control IgG group (Fig. 6e). To determine whether the activation of the MAPK/ERK pathway was driven by ACM and to identify the correlation with periostin expression, we used ACM both with and without periostin NeuAbs to treat TNBCs to examine the activation of the MAPK/ERK pathway. The results indicated that the MAPK/ERK pathway of the TNBCs was significantly activated by ACM stimulation, but the presence of periostin NeuAbs inhibited the activation of the MAPK/ERK signaling pathway (Supplemental Figure S1). To clarify whether the MAPK/ERK signaling pathway is involved in periostin**–**mediated cellular processes, a specific inhibitor of the MAPK/ERK pathway, U0126**–**EtOH, was exogenously added to the culture medium of MDA**–**MB**–**231 cells treated with rhPeriostin. Western blotting analysis results showed that U0126**–**EtOH suppressed the rhPeriostin**–**induced increase in the p**–**ERK1/2 level (Fig. 6f). Moreover, when ERK signaling was inhibited by U0126**–**EtOH, the rhPeriostin**–**induced increases in the expression of MMPs and stemness**–**related molecules were significantly suppressed (Fig. 6g–h), and there was reduced rhPeriostin**–**induced PTX chemoresistance in MDA**–**MB**–**231 cells (Fig. 6i). These results reveal that periostin in TNBCs mediates extracellular matrix degradation, stemness, and chemoresistance via the MAPK/ERK signaling pathway.

### High POSTN expression correlates with poor survival in patients with TNBC

**Fig. 7:**
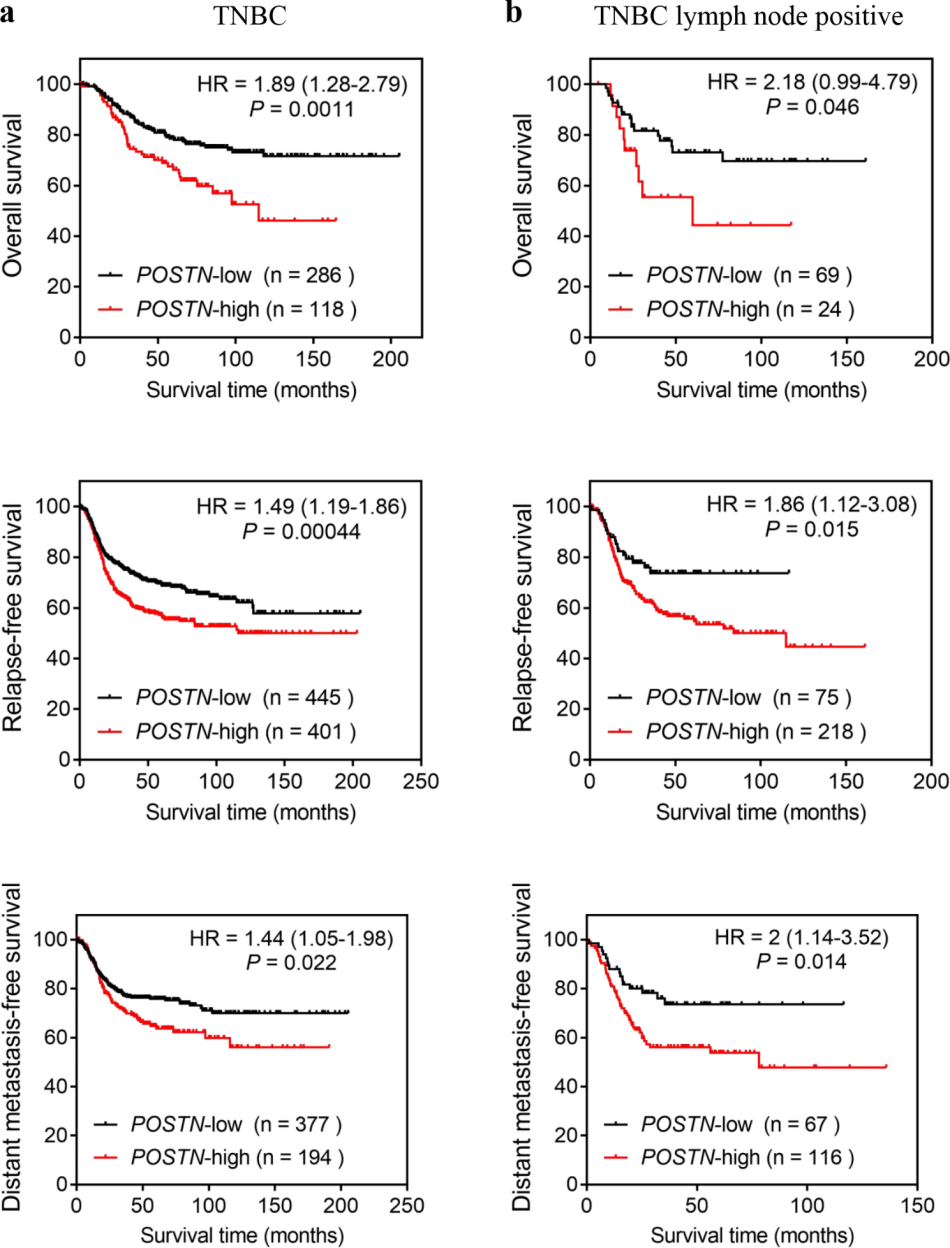
High POSTN expression correlates with poor survival in patients with TNBC. Kaplan–Meier curves of survival in TNBC patients, as calculated using TCGA data. The median expression value was used to separate the POSTN**–**low and POSTN**–**high groups based on the Kaplan**–**Meier curve. **a–b.** POSTN expression was assessed for its association with OS, RFS, and DMFS in TNBC patients. The expression groups were divided into high (red) and low (black). The survival times are shown in months, and HR and confidence intervals (in brackets) are shown for the gene

We further examined the relationship between the POSTN expression level and prognosis of patients with TNBC. The association between POSTN mRNA expression and survival in TNBC was analyzed using the Kaplan–Meier plotter online database. The median expression value was used to separate the POSTN**–**low and POSTN**–**high groups in the Kaplan–Meier curve. The Kaplan–Meier curve was presented with the hazard ratio (HR), 95% confidence intervals, and computed log rank *P* value. The results indicated that TNBC patients with high POSTN expression had significantly shorter OS, RFS, and DMFS than those with low POSTN expression (Fig. 7a). Additionally, the OS, RFS, and DMFS were significantly shorter in TNBC patients with positive lymph nodes who had high POSTN expression than in those who had low POSTN expression (Fig. 7b). Therefore, we speculate that POSTN plays a vital role in TNBC progression.

## Discussion

This study clearly demonstrated that ACM**–** and PA**–**induced periostin expression promotes extracellular matrix degradation, stemness, and PTX resistance in TNBCs via activation of the MAPK/ERK signaling pathway. Moreover, this study elucidated for the first time that periostin is the key protein secreted in TNBCs in response to a lipid environment, in addition to revealing the linkage between periostin and the MAPK/ERK signaling pathway and TNBC progression. Furthermore, the study indicated a novel mechanistic pathway describing how adipocytes and PA, as the predominant saturated fatty acid seen in obesity, contribute to TNBC progression.

Obesity has grown at an unprecedented rate in recent decades, becoming a major health concern worldwide [25]. Accumulating evidence suggests a strong link between obesity and higher morbidity and mortality from a variety of cancers, including prostate cancer, hepatocellular carcinoma, and breast cancer [7, 26]. In recent years, an increasing number of epidemiological studies have demonstrated the important role of obesity in breast cancer progression [27]. Adipocytes are vital regulators of the breast tumor microenvironment that facilitate disease progression by acting either directly or indirectly on cancer cells [28]. While PA is a very common saturated fatty acid found in many dietary fats, including lard, butter, peanut oil, tallow, and olive oil [29], it can also be synthesized endogenously, ingested exogenously, and participate in cancer development [30]. However, the interaction and underlying mechanisms between adipocytes and PA and breast cancer cells at the microenvironment level remain unclear [7].

Our results showed that periostin expression and secretion were significantly elevated in TNBCs treated with ACM and PA, while the inhibition of periostin expression or neutralization of periostin with periostin**–**neutralizing antibodies suppressed the degradation of the extracellular matrix, stemness, and chemoresistance promoted by ACM and PA in TNBCs. Interestingly, aside from TNBCs, we also found that ACM induced the elevated expression of periostin in other hormone receptor**–**positive breast cancer cells (such as T47D and ZR7530), as detailed in Supplementary Figure S2, indicating that reciprocal communication between adipocytes and breast cancer cells is very common. Furthermore, we investigated the pattern of POSTN mRNA expression in the breast cancer subclasses using UALCAN, which is a comprehensive, interactive web resource for analyzing cancer omics data. The results revealed that POSTN expression was significantly higher in breast cancer tissues than in normal breast tissues. In the subclasses of breast cancer, POSTN expression was highest in the luminal subtype of breast cancer, followed by the HER2**–**positive and TNBC subtypes (Supplementary Figure S3), suggesting that periostin plays a critical role in breast cancer. Prior studies have shown that the aberrant expression of periostin may be related to the malignant progression of tumors [31]; however, the biological function of periostin in the breast cancer and adipocyte microenvironment remains unclear. Our study suggested that the elevated expression of periostin activated the MAPK/ERK signaling pathway, which induced extracellular matrix degradation, enhanced stemness, and chemoresistance in TNBCs. The MAPK/ERK signaling pathway has been reported to be involved in a variety of biological events, including metabolic reprogramming, cell proliferation, survival, and differentiation, while dysregulation of this pathway is a very common event in various human malignancies [32, 33]. Finally, our study demonstrated a strong association between high POSTN expression and shorter survival rates in patients with TNBC.

### Study strengths and limitations

The mechanisms of periostin involved in the interaction between TNBCs and adipocytes are being for the first time in this study. The results demonstrated that ACM and PA upregulated the expression of periostin in TNBCs, leading to increased expression of MMPs and stemness**–**related molecules to promote extracellular matrix degradation and enhance stemness. Importantly, upregulated periostin significantly attenuated the efficacy of PTX on TNBCs, suggesting that periostin has an impact on chemoresistance. Nevertheless, it is important to note that this study had several limitations. First, the ACM contained a complicated composition, including a wide range of secretory factors, such as cytokines, growth factors, hormones, and extracellular matrix**–**processing proteases, which are known as adipose**–**derived secreted factors or adipokines released by adipocytes [34, 35]. In this study, we used 100% ACM to perform the in vitro experiments and did not focus on the specific components of the ACM that elevated the expression of MMPs and stemness genes in TNBCs. Second, we did not detect the pattern of periostin protein expression in tissue samples from breast cancer patients. Third, we did not use an in vivo animal model to further support our cellular experiments. We intend to address these limitations by performing further experiments in future studies.

## Conclusions

This study clarifies the significance of the secreted protein periostin in mediating communication between TNBCs and the surrounding adipocytes within the tumor microenvironment. ACM**–** and PA**–**induced periostin expression promotes the extracellular matrix degradation, stemness, and chemoresistance of TNBCs by activating the MAPK/ERK signaling pathway. Targeting periostin can significantly suppress TNBC progression. Therefore, a better understanding of the underlying molecular mechanisms behind the interaction between adipocytes and breast cancer cells may lead to the identification of new molecular targeted agents. Moreover, targeting the tumor microenvironment may represent an attractive strategy for preventing breast cancer progression.

### Electronic supplementary material

Below is the link to the electronic supplementary material.

Supplementary Material 1

## Data Availability

All data generated or analyzed during this study are included in this article.

## Abbreviations

TNBC: Triple**–**negative breast cancer
ACM: Adipogenic conditioned medium
PA: Palmitic acid
MMPs: Matrix metalloproteinases
ER: Estrogen receptor
PR: Progesterone receptor
HER2: Human epidermal growth factor receptor 2
ATCC: American Type Culture Collection
RT**–**qPCR: Real**–**time quantitative polymerase chain reaction
ELISA: Enzyme**–**linked immunosorbent assay
GO: Gene Ontology
KEGG: Kyoto Encyclopedia of Genes and Genomes
SD: Standard deviation
ANOVA: One-way analysis of variance
shPOSTN: POSTN**–**specific shRNA
rhPeriostin: Recombinant human periostin protein
NeuAbs: Neutralizing antibodies
PTX: Paclitaxel
RNA**–**seq: RNA sequencing
BP: Biological processes
p**–**P38: Phosphorylation of p38
p**–**ERK1/2: Phosphorylation of ERK1/2
OS: Overall survival
RFS: Relapse**–**free survival
DMFS: Distant metastasis**–**free survival
HR: Hazard ratios
