# Supplementary material for: Periostin drives extracellular matrix degradation, stemness, and chemoresistance by activating the MAPK/ERK signaling pathway in triple–negative breast cancer cells
Source: Lipids Health Dis. 2023 Sep 16;22:153. doi: 10.1186/s12944-023-01912-1 (PMC10504790; doi:10.1186/s12944-023-01912-1)
Supplement: Supplementary file 1 — Supplementary Material 1 [file 12944_2023_1912_MOESM1_ESM.docx]

**Periostin drives extracellular matrix degradation, stemness, and chemoresistance by activating the MAPK/ERK signaling pathway in triple****–negative breast cancer cells**

Jinna Wu^*,1^, Jia Li^*,1^, Huiya Xu^*,2^, Ni Qiu^1^, Xiaojia Huang^1^, Hongsheng Li^#,1^

*^1^Department of Breast Oncology Surgery, Affiliated Cancer Hospital & Institute of Guangzhou Medical University, Guangzhou, 510095, China*

*^2^Department of Pathology, Sun Yat-sen Memorial Hospital, Sun Yat-sen University, Guangzhou 510120, China*

^*^These authors contributed equally to this work.

^#^Correspondence: Hongsheng Li, email:docli999@163.com


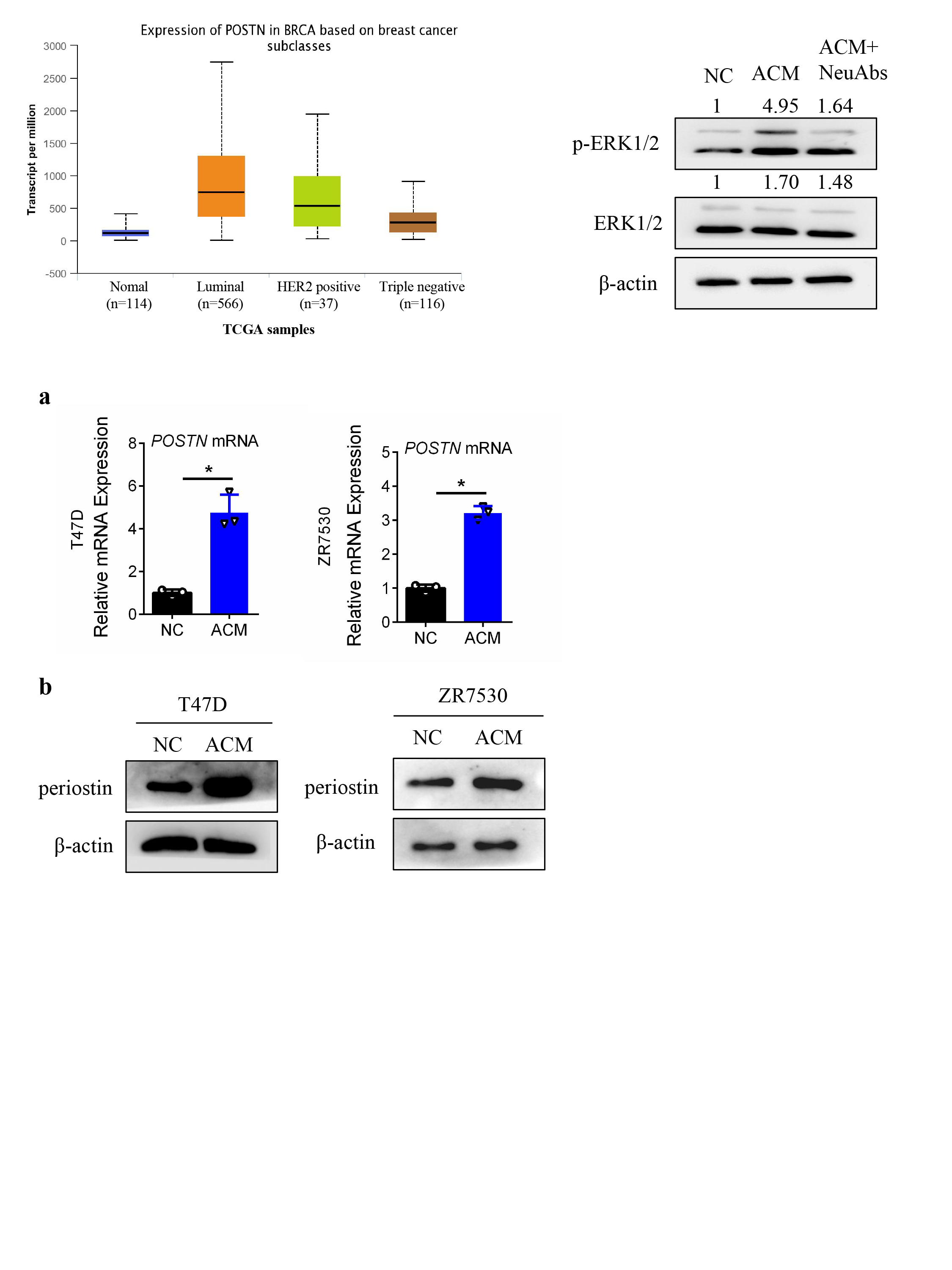


**Supplemental Figure S1:** Western blotting analysis of indicated protein level in MDA–MB–231 cells treated with NC or ACM with/without periostin NeuAbs for 24 h. The densitometry results of p–ERK1/2 (upper and bottom strips) were expressed as fold change in the protein levels when compared with NC–treated MDA–MB–231 cells after being normalized to β–actin. NC: negative control, ACM: adipogenic conditioned medium. NeuAbs: periostin neutralizing antibodies


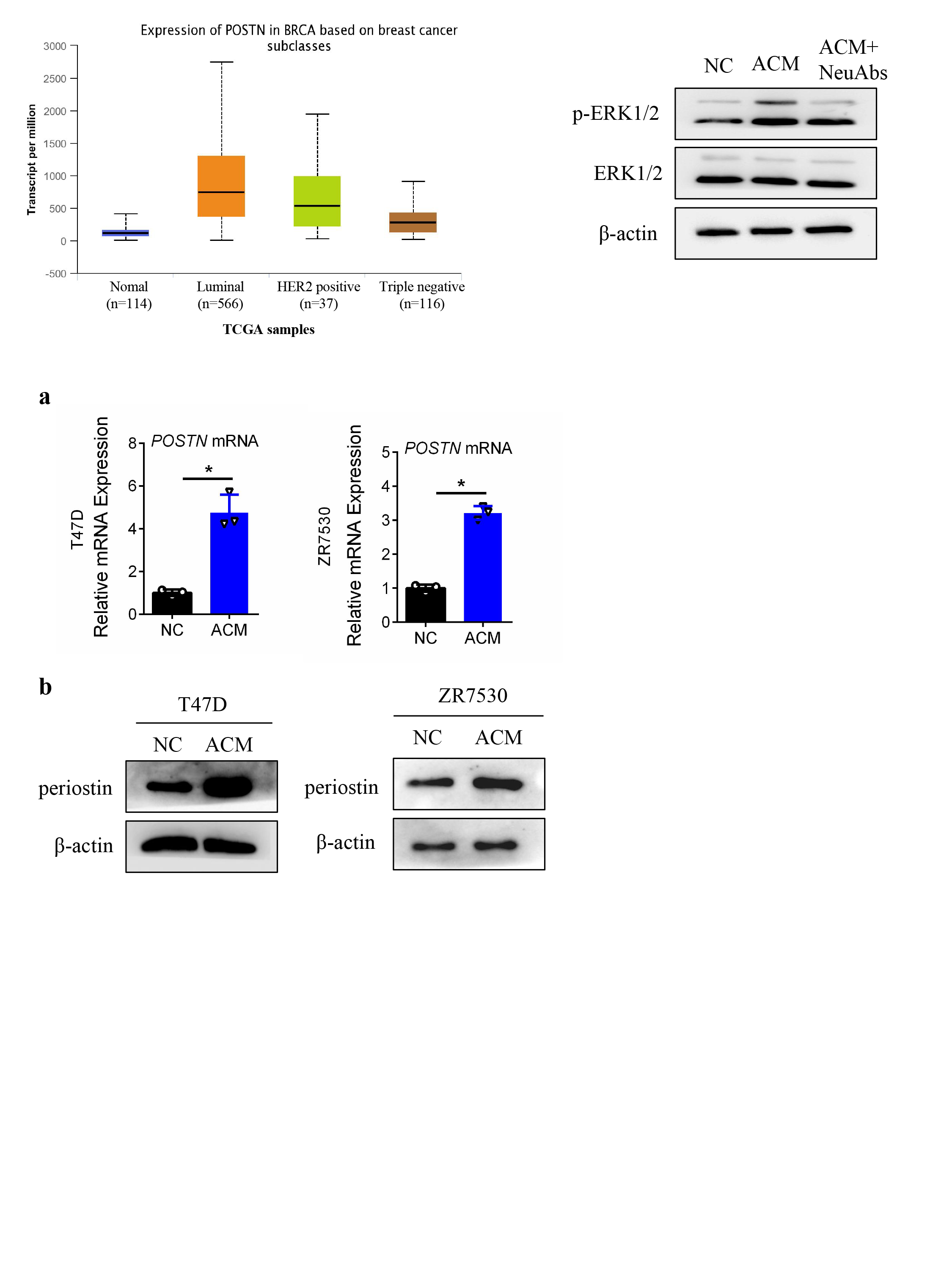


**Supplemental Figure S2:** **a.** POSTN mRNA expression in T47D and ZR7530 cells treated with NC or ACM for 24 h. **b.** Periostin expression level in T47D and ZR7530 cells treated with NC or ACM for 24 h. NC: negative control, ACM: adipogenic conditioned medium. **P* < 0.05.


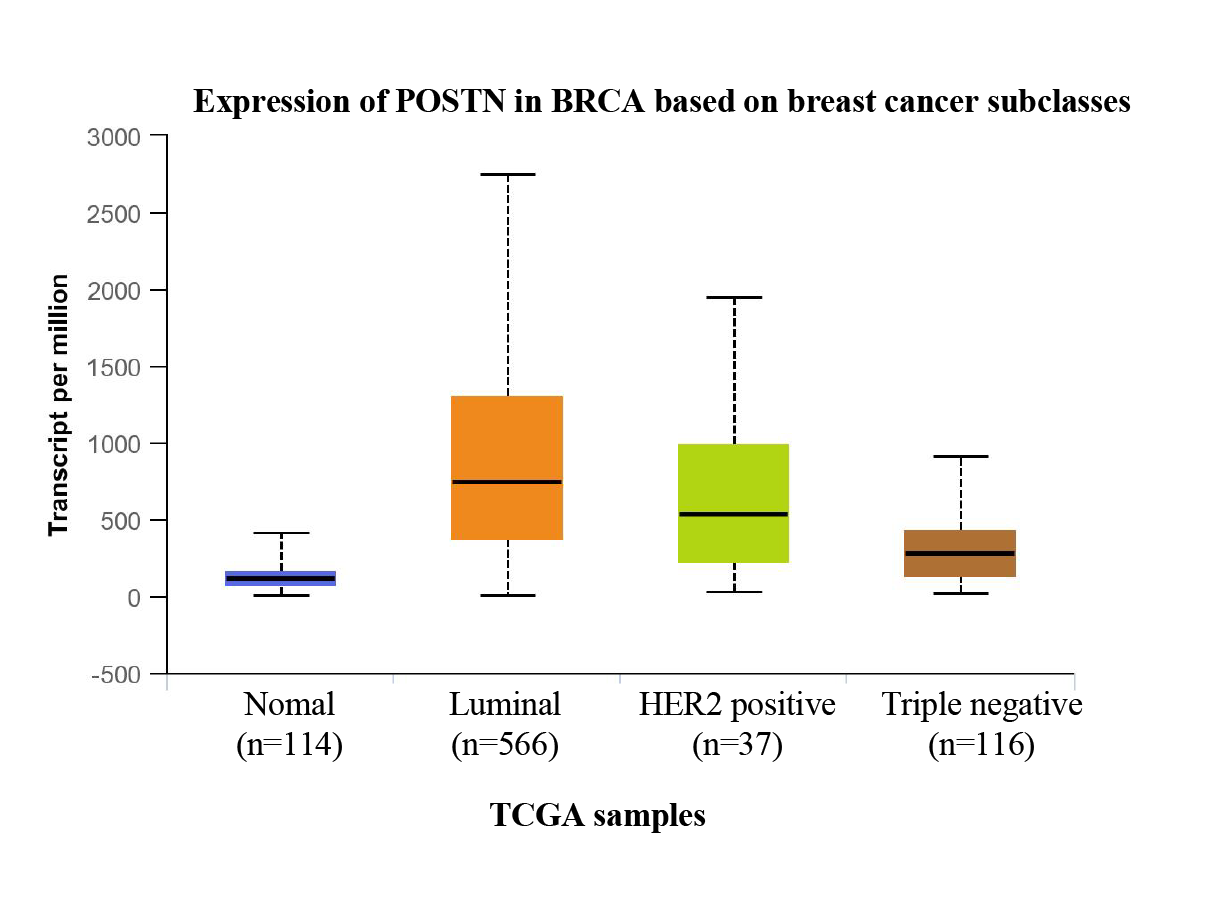


**Supplemental Figure S3:** Expression of POSTN in breast cancer based on different subclasses.

**Supplemental Table S1.** Clinical and pathological information of triple–negative breast cancer patients.

| Sample Number | Years | ER status | PR status | HER2 status | T | N | M |
| --- | --- | --- | --- | --- | --- | --- | --- |
| 1 | 51 | - | - | - | 2 | 0 | 0 |
| 2 | 50 | 1% | - | - | 4 | 2 | 0 |
| 3 | 68 | - | - | 1+ | 3 | 0 | 0 |
| 4 | 55 | - | - | - | 2 | 0 | 0 |
| 5 | 53 | - | - | - | 4 | 0 | 0 |

ER: estrogen receptor; PR: progesterone receptor

TNM classification, T: tumor; N: node; M: metastasis
